# Pan-Cancer Prognostic, Immunity, Stemness, and Anticancer Drug Sensitivity Characterization of N6-Methyladenosine RNA Modification Regulators in Human Cancers

**DOI:** 10.3389/fmolb.2021.644620

**Published:** 2021-06-04

**Authors:** Rui Li, Yun-Hong Yin, Xiu-Li Ji, Xiao Liu, Jian-Ping Li, Yi-Qing Qu

**Affiliations:** ^1^ Department of Pulmonary and Critical Care Medicine, Qilu Hospital, Cheeloo College of Medicine, Shandong University, Jinan, China; ^2^ Department of Pulmonary and Critical Care Medicine, Qilu Hospital of Shandong University, Jinan, China; ^3^ Department of Pulmonary Disease, Traditional Chinese Medicine Hospital of Jinan, Jinan, China

**Keywords:** pan-cancer analysis, m6A regulators, tumor immune microenvironment, drug sensitivity, overall survival

## Abstract

N6-methyladenosine RNA modification plays a significant role in the progression of multiple tumorigenesis. Our study identified the imperative role of m6A regulators in the tumor immune microenvironment, survival, stemness score, and anticancer drug sensitivity of pan-cancer. The Wilcox test was to identify the differential expression between 17 m6A regulators across 33 TCGA cancer types and their normal tissues from UCSC Xena GDC pan-cancer. Survival analysis of m6A-related regulators in 33 TCGA cancer types was identified using the “survival” and “survminer” package. The Spearman correlation test and Pearson correlation test were used to identify the correlation relationship between m6A regulators expression and tumor microenvironment, tumor stem cell score, and drug sensitivity of anticancer drugs. ConsensusPathDB was used for exploring m6A regulators functional enrichment. The 17 (METTL3, WTAP, METTL14, RBM15, RBM15B, VIRMA, HNRNPC, HNRNPA2B1, YTHDC1, ZC3H13, YTHDF1, YTHDC2, YTHDF2, IGF2BP3, IGF2BP1, FTO, and ALKBH5) m6A regulators were differentially expressed in 18 TCGA cancer types and adjacent normal tissues. Correlation analysis indicated that the relationship between the expression of 17 m6A regulators and tumor microenvironment indicated that the higher expression of m6A regulators, the higher the degree of tumor stem cells. The anticancer drug sensitivity analysis indicated that ZC3H13 expression had a positive relationship with anticancer drugs such as selumetinib, dabrafenib, cobimetinib, trametinib, and hypothemycin (*p* < 0.001). YTHDF2 expression was significantly negatively correlated with the anticancer drug dasatinib (*p* < 0.001). The pan-cancer immune subtype analysis showed that the 17 m6A regulators were significantly different in immune subtype C1 (wound healing), C3 (inflammatory), C2 (IFN-gamma dominant), C5 (immunological quiet), C4 (lymphocyte depleted), and C6 (TGF-beta dominant) (*p* < 0.001). Our study provides a comprehensive insight for revealing the significant role of m6A regulators in the tumor immune microenvironment, stemness score, and anticancer drug sensitivity of human cancers.

## Introduction

N6 adenosine methylation (m6A), as the most common type of RNA modification, is regulated by three types of regulators, including m6A-binding proteins (“readers”), methyltransferases (“writers”), and demethylases (“erasers”), which play a significant role in the progression and development of cancer (Choe et al., 2018; Yang et al., 2018). The methyltransferases complex includes METTL3, METTL14, WTAP, RBM15, RBM15B, VIRMA, and ZC3H13 (Wang et al., 2017). The m6A-binding proteins contain HNRNPA2B1, HNRNPC, IGF2BP1, IGF2BP3, YTHDF1, YTHDF2, YTHDC1, and YTHDC2 (Roundtree et al., 2017; Yang et al., 2018). The demethylases include FTO and ALKBH5 (Lan et al., 2019). Recently, m6A regulators related to onco-immunogenic characteristics and prognosis among 33 TCGA cancer types were reported (Chen et al., 2020). A signature of seven m6A regulators predicts the survival rate of patients in multiple human cancers (Kandimalla et al., 2019). A recent study analyzed the clinical relevance and molecular alterations of m6A regulators across 33 cancer types (Li et al., 2019). Differential expression, tumor microenvironment, drug sensitivity, Cox, and immune subtype analysis of m6A regulators among 33 TCGA cancer types were still lacking.

M6A modification regulators play a significant role in the progression and prognosis of lung adenocarcinoma (Choe et al., 2018; Li et al., 2019; Sheng et al., 2020). METTL3, as an RNA methyltransferase, promotes the translation of certain mRNAs, incorporating the Hippo pathway effector TAZ and epidermal growth factor receptor (EGFR) among human cancer cells, which promotes growth, invasion, and survival in human lung cancer cells (Lin et al., 2016). YTHDF1 as a m6A-binding protein plays a vital role in pathogenesis and hypoxia adaptation of non-small cell lung cancer (NSCLC) (Shi et al., 2019). A study demonstrated that m6A demethylase (“eraser”) ALKBH5 inhibits LATS2/miR-107-mediated YAP activity and inhibits tumor proliferation and metastasis through decreasing the m6A-binding proteins YTHDFs-associated YAP expression in NSCLC (Jin et al., 2020). Lung cancer is still the most common cause of cancer-associated death worldwide (Gansler et al., 2010). Although new treatment has been improved in NSCLC, the 5-year survival rate was still not optimistic (Siegel et al., 2017).

In our study, we first analyzed the expression level of reported 17 (METTL3, METTL14, WTAP, RBM15, RBM15B, VIRMA, ZC3H13, HNRNPA2B1, HNRNPC, YTHDF1, YTHDF2, YTHDC1, YTHDC2, IGF2BP1, IGF2BP3, FTO, and ALKBH5) m6A-related regulators across 33 cancer types and analyzed the differential expression, tumor microenvironment, immune subtype, and drug sensitivity of m6A-related regulators of 33 cancer types. Furthermore, the association between m6A-related regulators, tumor stem cell score, and immune subtype of NSCLC was analyzed, which paved a novel road for revealing the new mechanisms and therapeutic targets of m6A regulators in pan-cancer and NSCLC.

## Materials and Methods

### Pan-Cancer Transcriptome Expression Data, Immunophenotype Data, and Stem Cell Score Data Download

We used the UCSC Xena (https://xenabrowser.net/) to download the 33 TCGA GDC pan-cancer (ACC, adrenocortical carcinoma; BLCA, bladder urothelial carcinoma; BRCA, breast invasive carcinoma; CESC, cervical squamous cell carcinoma and endocervical adenocarcinoma; CHOL, cholangiocarcinoma; COAD, colon adenocarcinoma; DLBC, lymphoid neoplasm diffuse large B-cell lymphoma; ESCA, esophageal carcinoma; GBM, glioblastoma multiforme; HNSC, head and neck squamous cell carcinoma; KICH, kidney chromophobe; KIRC, kidney renal clear cell carcinoma; KIRP, kidney renal papillary cell carcinoma; LAML, acute myeloid leukemia; LGG, brain lower grade glioma; LUSC, lung squamous cell carcinoma; LIHC, liver hepatocellular carcinoma; LUAD, lung adenocarcinoma; MESO, mesothelioma; OV, ovarian serous cystadenocarcinoma; PRAD, prostate adenocarcinoma; PAAD, pancreatic adenocarcinoma; PCPG, pheochromocytoma and paraganglioma; READ, rectum adenocarcinoma; STAD, stomach adenocarcinoma; SARC, sarcoma; SKCM, skin cutaneous melanoma; TGCT, testicular germ cell tumors; THCA, thyroid carcinoma; THYM, thymoma; UCEC, uterine corpus endometrial carcinoma; UVM, uveal melanoma; and UCS, uterine carcinosarcoma) transcription expression data and TCGA pan-cancer phenotype-immune subtype data and stemness score (based on DNA methylation) and stemness score (based on RNA expression) data.

### Gene Expression Analysis and Differential Expression Analysis

We used the Ensembl (https://asia.ensembl.org/index.html) to obtain the gene symbol of transcription expression data. Then, we deleted the normal data and kept the transcription data of 33 TCGA cancers to show a box plot of the expression levels of 17 m6A regulators (METTL3, WTAP, METTL14, RBM15, RBM15B, VIRMA, HNRNPC, HNRNPA2B1, YTHDC1, ZC3H13, YTHDC2, YTHDF2, IGF2BP3, IGF2BP1, FTO, YTHDF1, and ALKBH5) in 33 tumors. Eventually, comparison of gene expression between normal and tumors was statistically performed in 18 cancer types, which had more than five associated adjacent normal tissues using linear mixed effects models (Yu et al., 2020; Zhang et al., 2020). Finally, we analyzed the differential expression level of 17 m6A regulators in 18 tumors (BLCA, CHOL, BRCA, ESCA, COAD, HNSC, GBM, KICH, LIHC, KIRC, KIRP, LUAD, PRAD, LUSC, STAD, READ, THCA, and UCEC). We used the Wilcox test to analyze the difference between adjacent normal and tumor tissues. A difference of *p*-value less than 0.05 was regarded statistically significant.

### Survival Analysis of Expression of m6A Regulators

Survival analysis of m6A-related regulators was used for the “survival” and “survminer” R package. A difference of *p* less than 0.05 was statistically significant.

### Functional Enrichment Analysis of m6A-Related Regulators

We used the ConsensusPathDB database (http://cpdb.molgen.mpg.de/) to perform functional enrichment analysis of m6A-related regulators. ConsensusPathDB-human is a comprehensive database, which integrates interaction networks in *Homo sapiens* including gene regulatory, genetic, protein–protein, signaling interactions, and biochemical pathways (Kamburov et al., 2011; Kamburov et al., 2013).

### M6A Regulators Correlation Analysis

To further identify the correlation relationship of m6A regulators, we conducted the m6A regulators correlation analysis. We used the “corrplot” R package to further perform the correlation analysis of m6A regulators. The association coefficient is greater than 0, which means there is a positive correlation between m6A-related regulators and less than 0 means negative correlation.

### Cox and Immune Subtype Analysis

We downloaded the 33 TCGA pan-cancer transcription expression and survival data to perform the Cox analysis to verify whether 17 m6A regulators expression related to the survival of patients. We used the “limma,” “ggplot2,” and “reshape2” R package to perform the immune subtype analysis of 17 m6A-related genes. The *p*-value < 0.05 was statistically significant.

### Correlation Analysis of the Tumor Microenvironment in 33 TCGA Pan-Cancers

We used the “estimate” R package and “limma” R package to obtain the immune score, stromal score, and estimate score of 33 TCGA tumor samples. Then, we intersected transcription gene expression data with estimate score of 33 TCGA cancer samples to perform the Spearman correlation test. Correlation analysis between 17 m6A-related genes and estimate score of 33 TCGA tumors was performed. We intersected transcription gene expression data with stemness score (RNA expression–based) (RNAss) to conduct the Spearman correlation test. We intersected transcription gene expression data with stemness score (DNA methylation–based) (DNAss) to perform the Spearman correlation test. Finally, we obtained the correlation relationship between 17 m6A regulators expression and RNAss/DNAss of 33 TCGA tumors.

### Drug Sensitivity Analysis

We used the CellMiner database (https://discover.nci.nih.gov/cellminer/home.do) to download the same sample of gene expression and drug sensitivity data and then filtered drug sensitivity data after clinical laboratory verification and FDA standard certification. Next, we combined the 17 m6A-related gene expression with drug sensitivity data to perform the Pearson correlation test. Eventually, the correlation relationship between m6A-related gene expression and drug sensitivity was obtained.

### Immune Subtype, Clinical Characteristic, and Tumor Microenvironment Analysis of Lung Adenocarcinoma and Lung Squamous Cell Carcinoma

To further identify the relationship between the m6A regulators expression in LUAD and LUSC and immune subtype, clinical characteristics, and LUAD and LUSC microenvironment, we conducted the Kruskal test to perform the differential analysis of immune subtype. We used the Kruskal test to conduct differential analysis of the pathological T stage, pathological TNM stage, and pathological N stage. We used the Wilcox test to perform a differential analysis of the pathological M stage. We first downloaded the expression matrix of 33 tumors and used the “estimate” R package to perform the tumor microenvironment analysis for obtaining the estimate score profile. Then, we used the Spearman correlation test to conduct the correlation analysis between m6A-related gene expression and LUAD and LUSC immune score, estimate score, DNAss, RNAss, and stromal score.

## Results

### The Expression Level of m6A Regulators From 18 TCGA Pan-Cancers

**FIGURE 1:**
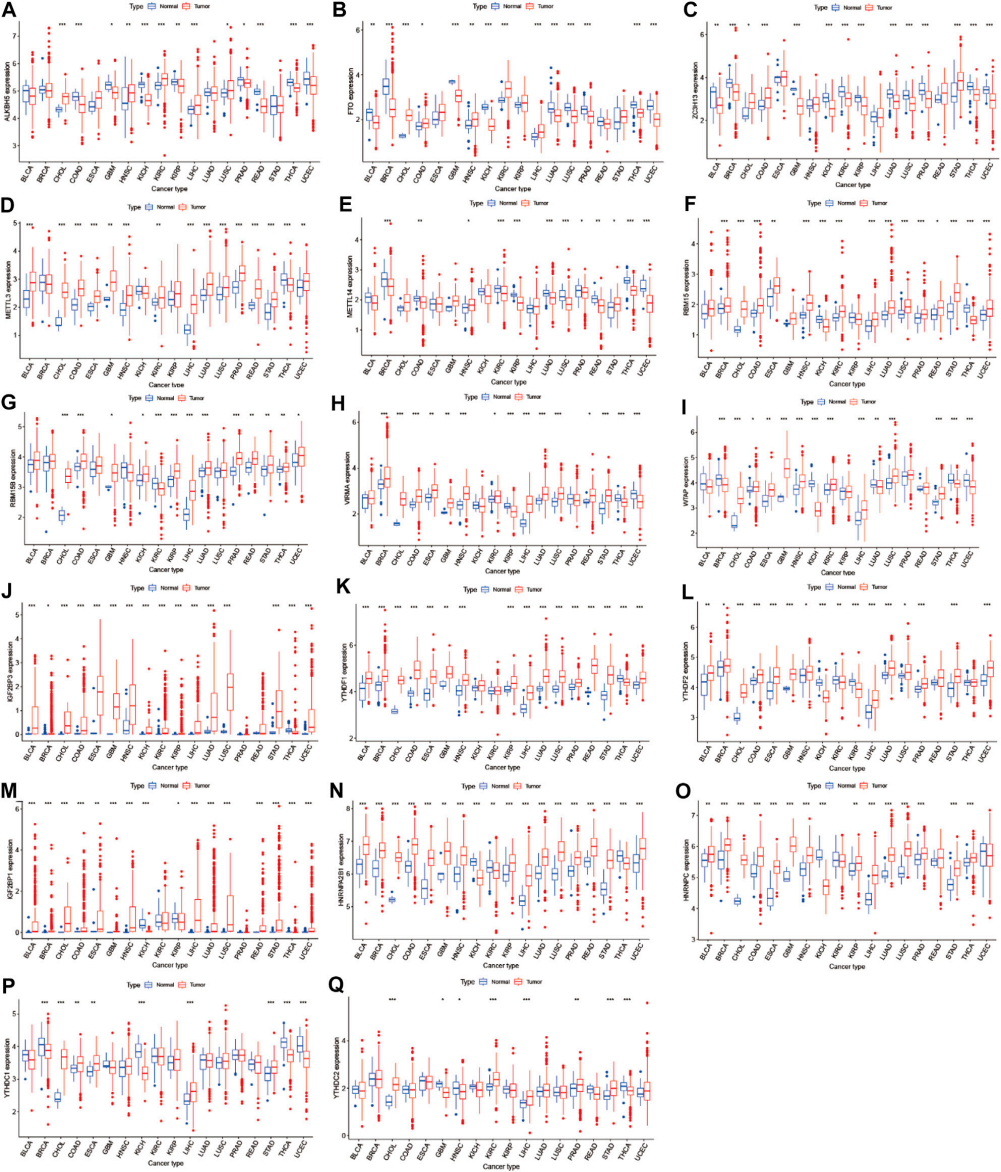
Boxplot of m6A regulators differential expression between cancer and adjacent normal tissues. The blue boxplots indicate the normal tissues. The red boxplots indicate the cancer tissues.

**FIGURE 2:**
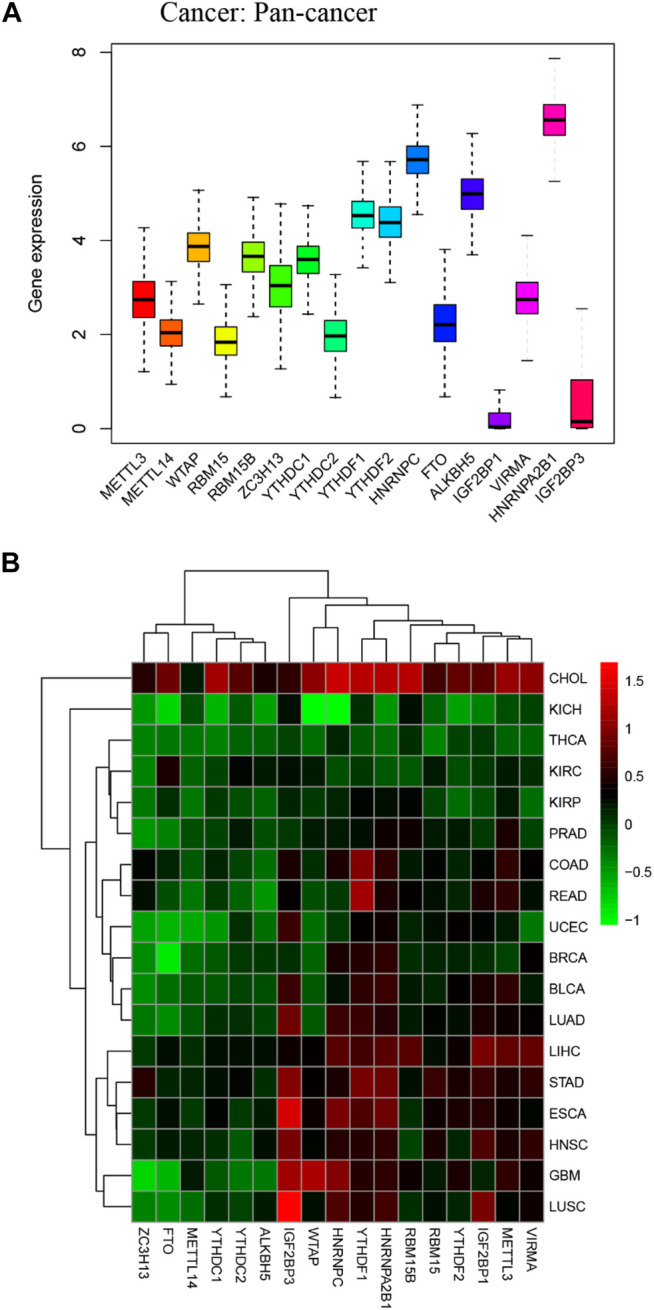
Boxplot and heatmap of 17 m6A regulators differential expression across 18 cancer types. **(A)** Boxplot of 17 m6A regulators expression in 18 TCGA cancer tissues. **(B)** The heatmap of 17 m6A regulators differential expression in 18 TCGA cancers. The red and green boxes indicate that the expression of m6A methylation regulators is high and low in correspondence cancer, respectively.

**FIGURE 3:**
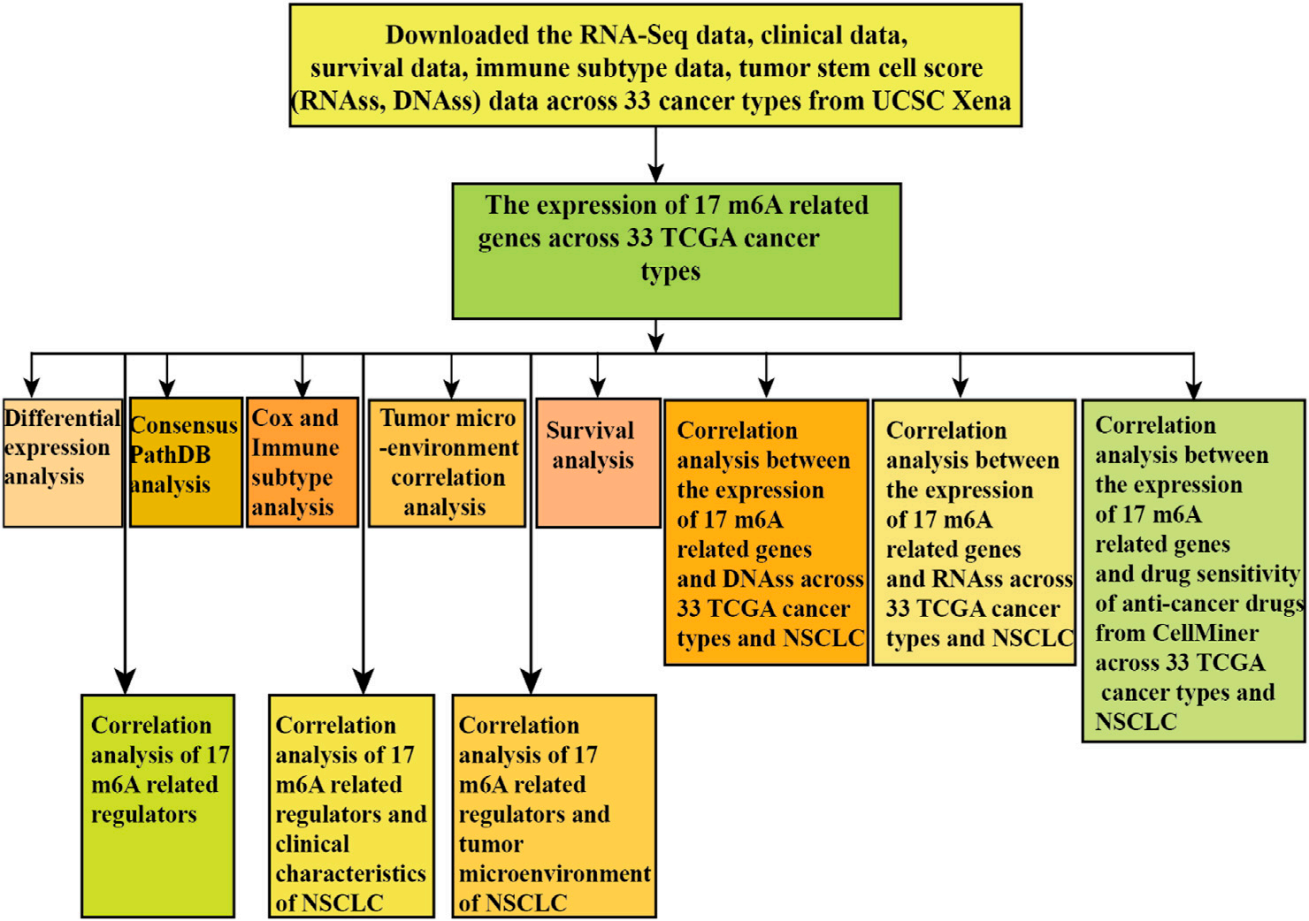
Flowchart of the pan-cancer analysis of m6A methylation regulators.

We obtained m6A regulators expression in TCGA database from 18 (BLCA, LUAD, COAD, BRCA, CHOL, HNSC, ESCA, GBM, KIRC, UCEC, READ, KICH, LIHC, KIRP, LUSC, THCA, PRAD, and STAD) cancers. Figure 1 shows that the differential expression level of m6A-demethylases (FTO and ALKBH5) (Figures 1A,B), m6A methyltransferases (ZC3H13, METTL3, METTL14, RBM15, RBM15B, VIRMA, and WTAP) (Figures 1C–I), and m6A-binding proteins (IGF2BP3, YTHDF1, YTHDF2, IGF2BP1, HNRNPA2B1, HNRNPC, YTHDC1, and YTHDC2) (Figures 1J–Q) between 18 TCGA tumors and their normal tissues (normal tissues more than five were selected as pan-cancer gene expression analysis). As we can see from Figure 1, there is no difference in the expression of ALKBH5, YTHDC1, and YTHDC2 in LUAD and adjacent normal tissues (*p* > 0.05). M6A-methyltransferases and demethylases were significantly different in the expression of LUAD and normal tissues. The expression levels of M6A-binding proteins (IGF2BP3, YTHDF1, YTHDF2, IGF2BP1, HNRNPA2B1, and HNRNPC) were significantly different between LUAD and normal tissues (*p* < 0.05). Figure 2A showed that the expression level of m6A regulators in 18 TCGA cancers. The expression levels of YTHDF1, YTHDF2, HNRNPC, and HNRNPA2B1 were higher in 18 TCGA pan-cancers, while the expression of IGF2BP1 and IGF2BP3 was lower in 18 TCGA pan-cancers. Figure 2B showed that the heatmap of the log (fold change (FC)) of m6A-related genes in 18 TCGA pan-cancers. Figure 2B indicated that the logFC of IGF2BP3, HNRNPC, and YTHDF1 was greater than 0, which indicated that the expression of IGF2BP3, HNRNPC, and YTHDF1 was highly expressed in LUAD tissues compared with adjacent non-LUAD tissues. The logFC of IGF2BP1 and IGF2BP3 was greater than 0, which indicated that IGF2BP1 and IGF2BP3 expression was highly expressed in LUSC tissues compared with adjacent non-LUSC tissues. The flow diagram of the whole study is shown in Figure 3.

### Pan-Cancer Survival Analysis of m6A-Related Regulators

**FIGURE 4:**
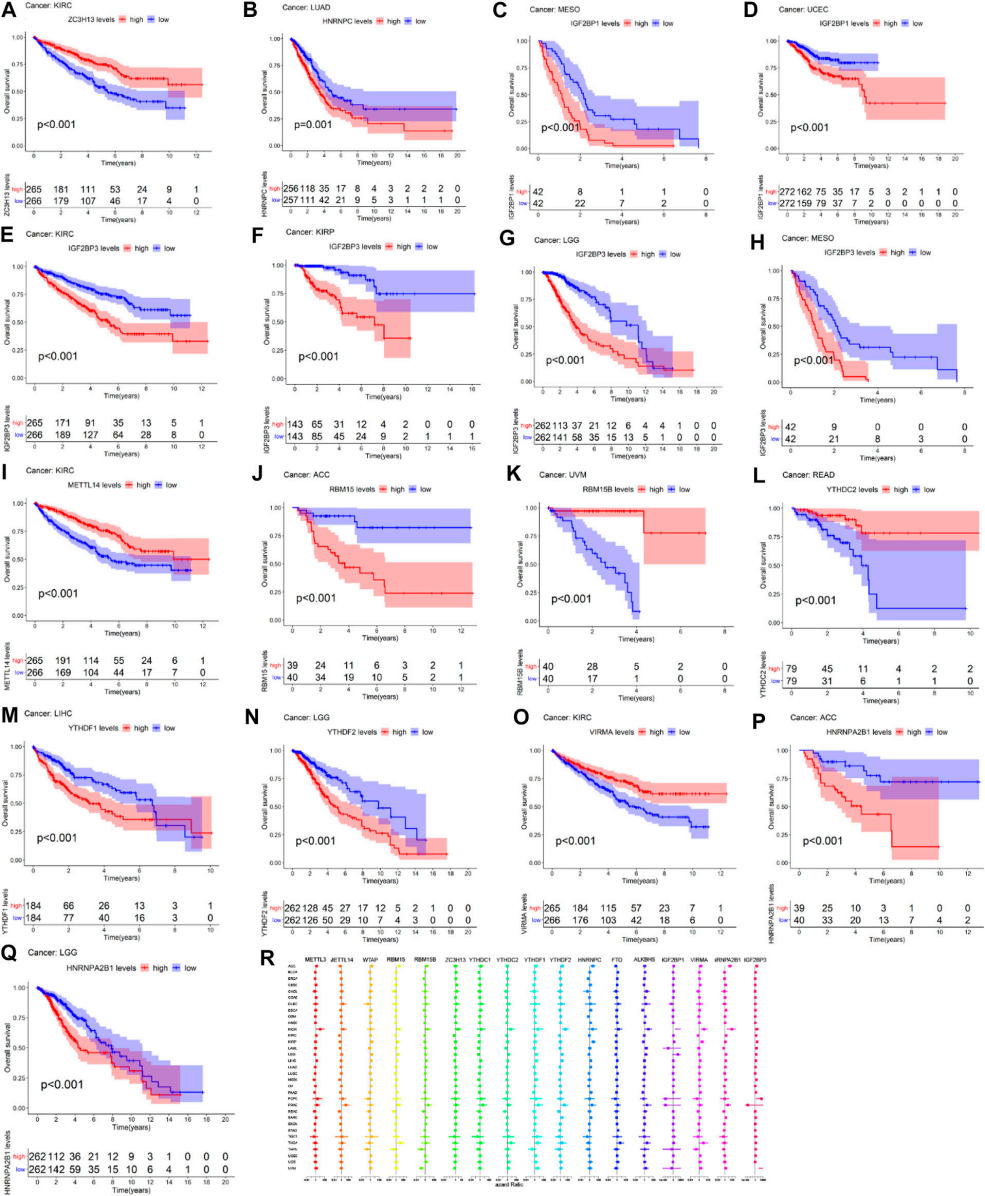
Survival analysis of m6A-related regulators across multiple cancer types. The red line in the photos indicates high expression and the blue line in the photos indicates low expression. (R) The forest map of hazard ratio of 17 m6A-related genes across 33 TCGA cancer types.

Figure 4 showed that the high expression of ZC3H13 had a better prognosis than the low expression of ZC3H13 in KIRC patients (*p* < 0.001). The high expression of HNRNPC had a poor prognosis than low expression of HNRNPC in LUAD patients (*p* = 0.001) (Figure 4B). The high expression of IFG2BP1 had a poor prognosis than the low expression of IGF2BP1 in MESO and UCEC patients (*p* < 0.001) (Figures 4C,D). The high expression level of IGF2BP3 had a poor prognosis than the low expression level of IGF2BP3 in KIRC, KIRP, LGG, and MESO patients (*p* < 0.001) (Figures 4E–H). The high expression of METTL14 had a better prognosis than the low expression of METTL14 in KIRC patients (*p* < 0.001) (Figure 4I). The low expression level of RBM15 had a better prognosis than the high expression level of RBM15 in patients with ACC (*p* < 0.001) (Figure 4J). The low expression level of RBM15B had a poor prognosis than the high expression level of RBM15B in UVM patients (*p* < 0.001) (Figure 4K). The high expression level of YTHDC2 significantly had a better prognosis than the low expression level of YTHDC2 in READ patients (*p* < 0.001) (Figure 4L). In LIHC patients, the low expression level of YTHDF1 had a better prognosis than the high expression level of YTHDF1(*p* < 0.001) (Figure 4M). In LGG patients, the high expression level of YTHDF2 significantly had a poor prognosis than the low expression of YTHDF2 (*p* < 0.001) (Figure 4N). The low expression of VIRMA had a poor prognosis than the high expression of VIRMA in KIRC patients (*p* < 0.001) (Figure 4O). The high expression of HNRNPA2B1 significantly had a poor prognosis than the low expression of HNRNPA2B1 in ACC and LGG patients (*p* < 0.001) (Figure 4P-Q). As we can see from Figure 4R, METTL3, METTL14, RBM15, YTHDC1, and YTHDF1 act as high-risk factors in KICH, PCPG, and PRAD patients. RBM15B acts as a high-risk factor in KICH and PRAD patients. ZC3H13 acts as a high-risk factor in KICH patients. YTHDC2 acts as high-risk factors in KICH, PRAD, TGCT, and THCA patients. YTHDF2, HNRNPC, and ALKBH5 serve as high-risk factors in KICH and PRAD patients. IGF2BP1 acts as high-risk factors in KICH and LGG patients; meanwhile, IGF2BP1 acts as low-risk factors in PCPG and PRAD patients.

### ConsensusPathDB Analysis of m6A Regulators

**FIGURE 5:**
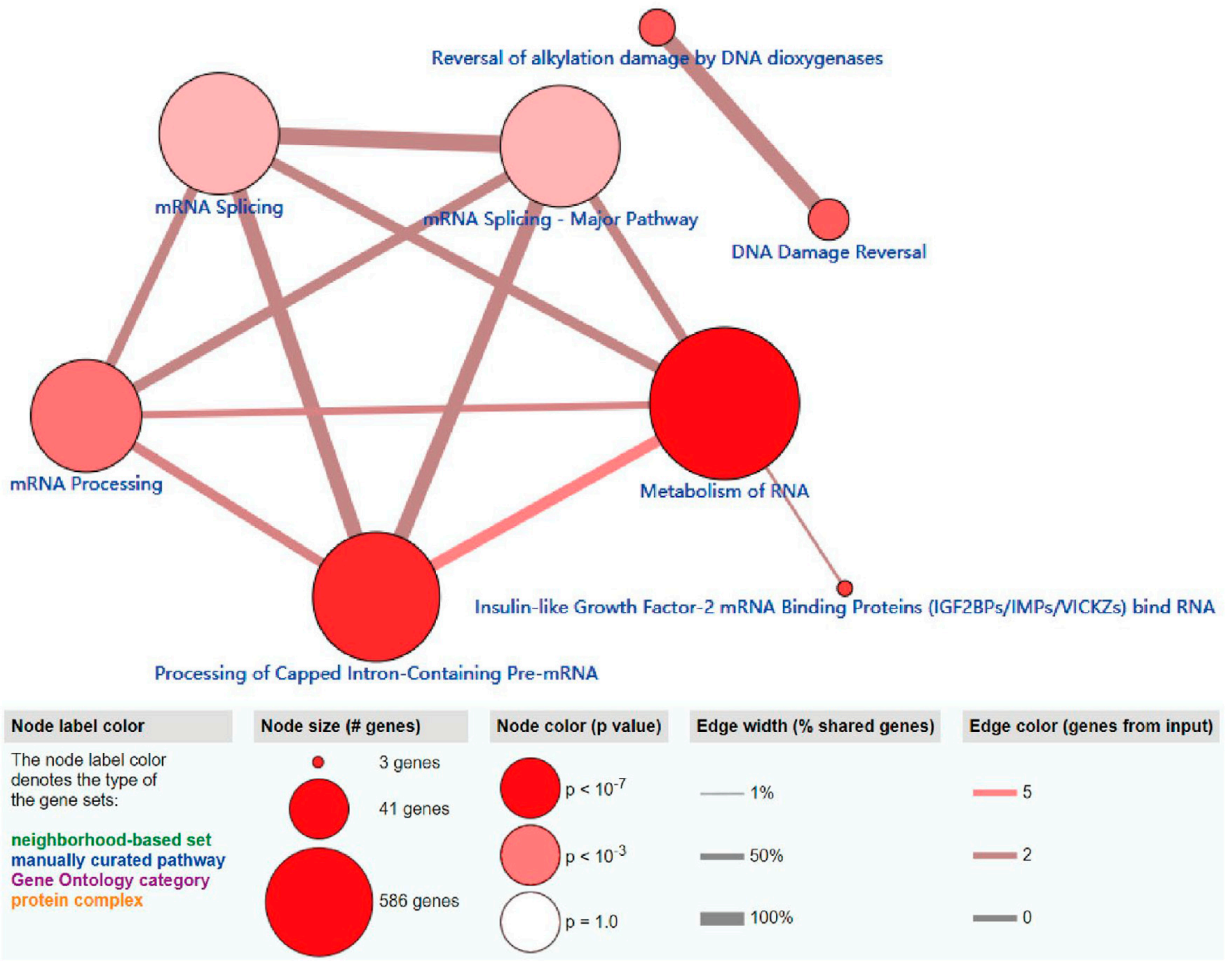
ConsensusPathDB analysis of functional enrichment of m6A-related regulators. The node label color denotes the type of the m6A-related regulators.

The ConsensusPathDB analysis indicated that m6A regulators were enriched in the metabolism of RNA, mRNA processing signaling pathway, and processing of capped intron-containing pre-mRNA, which indicated that m6A-related regulators participate in mRNA maturation processing and mRNA metabolism processing (Figure 5).

### Correlation and Cox, Tumor Immune Microenvironment, and Tumor Microenvironment Analysis of m6A-Associated Regulators in Pan-Cancer

**FIGURE 6:**
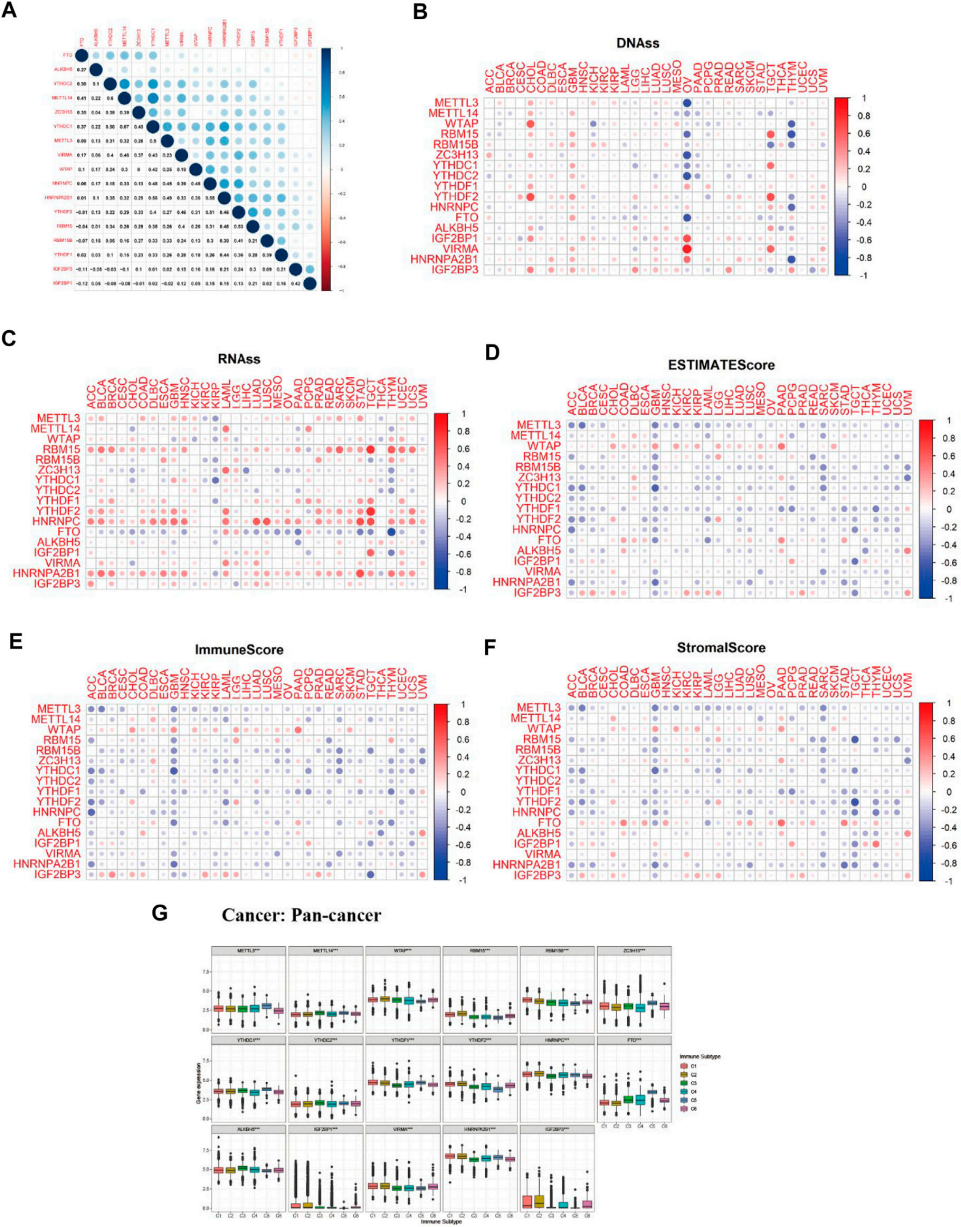
Correlation analysis between m6A-related regulators expression and pan-cancer immune microenvironment and pan-cancer microenvironment. **(A)** The blue and red dots indicate that m6A methylation regulators expression level had a negative and positive relationship, respectively. **(B)** The forest map of hazard ratio of 17 m6A-related genes across 33 TCGA cancer types. **(C,D)** The correlation relationship between 17 m6A regulators expression and tumor stem cell score (based on RNA expression and DNA methylation). **(E–G)** The correlation relationship among the expression of 17 m6A methylation regulators and estimate score, immune score, and stromal score. **(H)** The boxplot of m6A regulators differential expression in six pan-cancer immune subtypes.

M6A regulator correlation analysis indicated that m6A methyltransferase METTL14 expression was positively correlated with the expression of m6A-binding protein YTHDC1 (Cor = 0.67). The expression of m6A-binding protein YTHDC1 was positively correlated with the expression of HNRNPA2B1(Cor = 0.56). The expression of m6A demethylase FTO was negatively related to the expression of m6A-binding protein IGF2BP1 (Cor = −0.12) (Figure 6A). As we can see from Figures 6B,C, most of the expression of m6A regulators had positively correlated with the RNAss and DNAss of 33 TCGA cancers, which demonstrated that the higher expression of m6A regulators, higher the index of the tumor stemness score, the stronger the activity of tumor stem cells, and lower the degree of tumor differentiation. As we can see from Figures 6D–F, most of the expression of m6A regulators was significantly negatively correlated with immune score, stromal score, and estimate score, which indicated that the content of immune and stromal cells was low in 33 TCGA cancers, and the content of tumor cells was high in 33 TCGA cancers. Figure 6G showed that expression of m6A-related genes in pan-cancer was significantly different in immune subtype C1 (wound healing), C2 (IFN-gamma dominant), C3 (inflammatory), C4 (lymphocyte depleted), C5 (immunological quiet), and C6 (TGF-beta dominant) (*p* < 0.001).

### Correlation Analysis Between m6A-Related Gene Expression and drug Sensitivity

**FIGURE 7:**
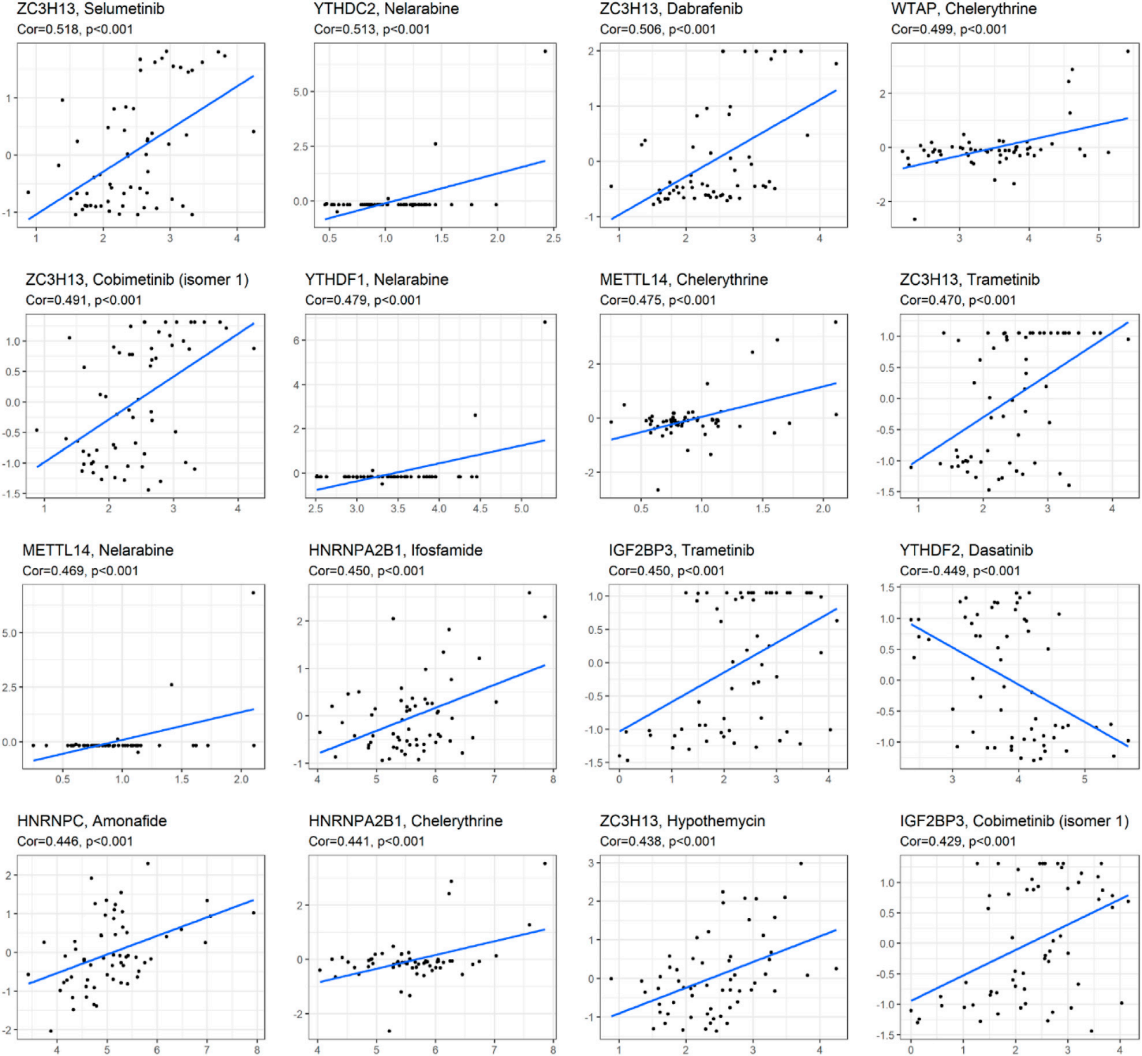
Correlation analysis between m6A methylation regulators and drug sensitivity of anticancer drugs in CellMiner. The top 16 statistical significance correlation relationship between m6A methylation regulators and drug sensitivity of anticancer drugs.

As we can see from Figure 7, the expression of ZC3H13 and IGF2BP3 had a positive relationship with the sensitivity of trametinib and cobimetinib (isomer 1) (*p* < 0.001). The higher the expression of YTHDC2 and METTL14, the stronger the drug sensitivity of nelarabine (*p* < 0.001). The higher the expression of YTHDF2, the weaker the drug sensitivity of dasatinib (*p* < 0.001). The expression of YTHDF1 had a significant positive relationship with the sensitivity of nelarabine (*p* < 0.001). The higher the expression of WTAP, METTL14, and HNRNPA2B1, the stronger the drug sensitivity of chelerythrine (*p* < 0.001). The higher the expression of HNRNPA2B1, the stronger the drug sensitivity of ifosfamide (*p* < 0.001). ZC3H13 expression had a significant positive relationship with the sensitivity of selumetinib, dabrafenib, and hypothemycin. The higher expression of HNRNPC, the stronger the drug sensitivity of amonafide (*p* < 0.001).

### Correlation Analysis of m6A-Related Gene Expression and Immune Subtype and Clinical Characteristics and Tumor Microenvironment of Lung Adenocarcinoma and Lung Squamous Cell Carcinoma

**FIGURE 8:**
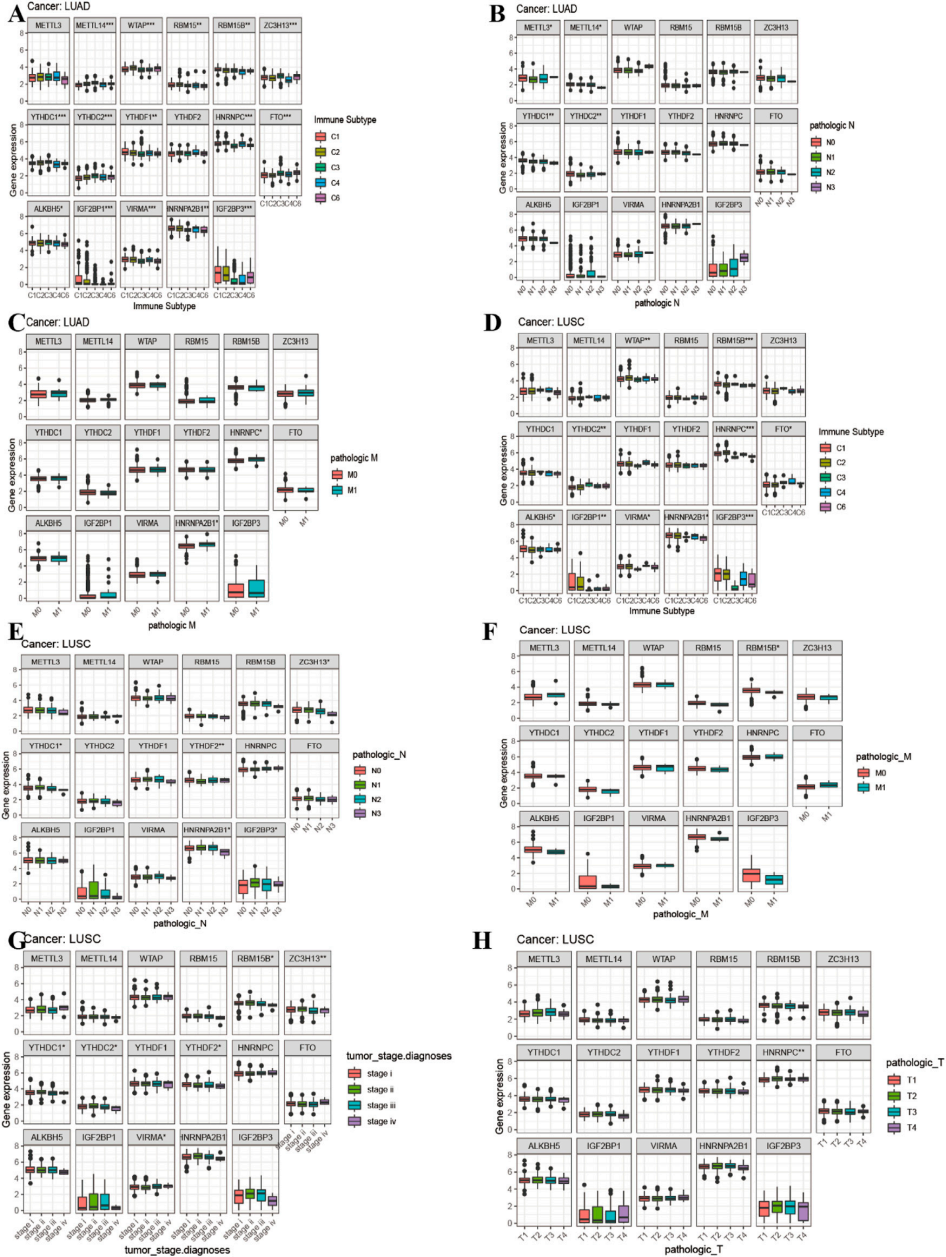
Correlation analysis between 17 m6A regulators expression and clinical characteristics in LUAD and LUSC. **(A)** Differences in m6A gene expression of LUAD between different immunophenotypes. **(B–C)** The boxplot of expression of 17 m6A-related genes and clinical features in LUAD. (D) Differences in m6A gene expression of LUSC between different immunophenotypes. (**E–H)** The boxplot of expression of 17 m6A-related genes and clinical features in LUSC.

**FIGURE 9:**
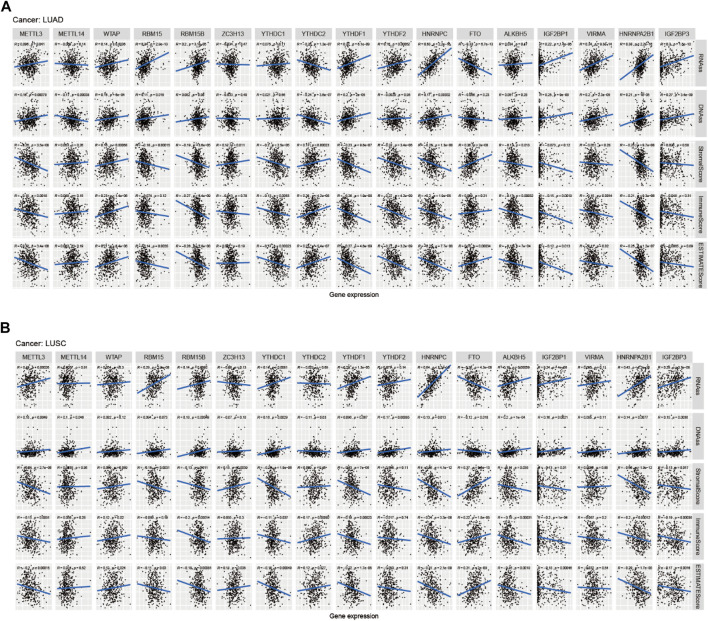
Correlation analysis of m6A regulators and LUAD, LUSC microenvironment, and stem cell scores. (A–B) The correlation relationship between m6A methylation regulators expression and LUAD, LUSC stem cell score, LUAD, and LUSC immune microenvironment.

**FIGURE 10:**
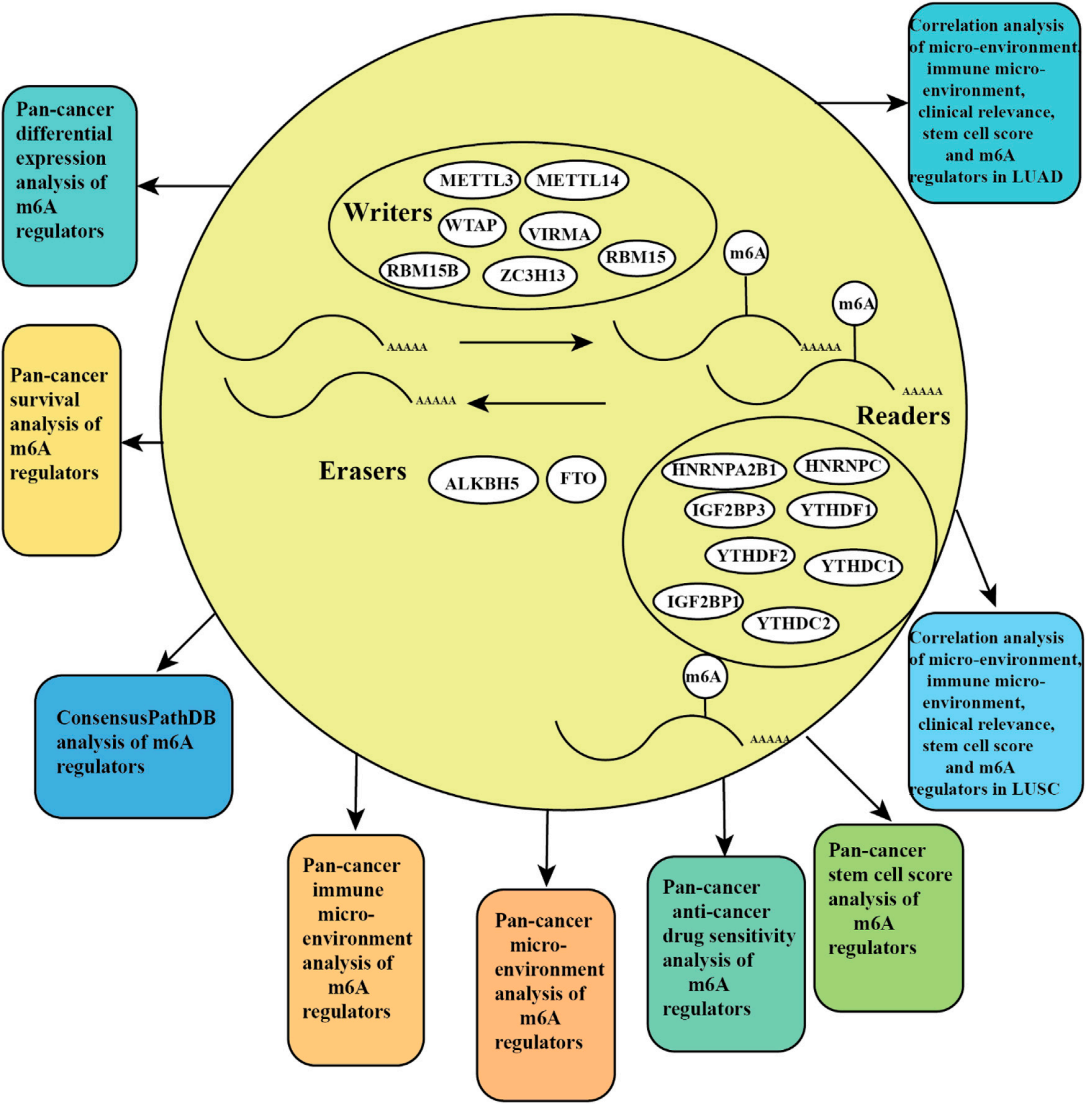
Pictorial representation of m6A regulation combined our findings.

Figure 8A showed that the expression of METTL14, WTAP, RBM15B, RBM15, YTHDC2, ZC3H13, YTHDC1, YTHDF1, FTO, HNRNPC, ALKBH5, IGF2BP1, VIRMA, HNRNPA2B1, and IGF2BP3 in LUAD was significantly different in immune type C1 (wound healing), C3 (inflammatory), C2 (IFN-gamma dominant), C4 (lymphocyte depleted), and C6 (TGF-beta dominant). Figure 8B showed that the expression of METTL3, METTL14, YTHDC1, and YTHDC2 in LUAD patients was significantly different in pathological N0/N1/N2/N3 stage. Figure 8C indicated that HNRNPA2B1 expression in LUAD was significantly different in the pathological M0/M1 stage. Figure 8D indicated that expression levels of WTAP, RBM15B, YTHDC2, HNRNPC, FTO, ALKBH5, IGF2BP1, VIRMA, HNRNPA2B1, and IGF2BP3 in LUSC patients were significantly different in immune type C1 (wound healing), C4 (lymphocyte depleted), C3 (inflammatory), C2 (IFN-gamma dominant), and C6 (TGF-beta dominant). Figure 8E showed that ZC3H13, YTHDC1, YTHDF2, HNRNPA2B1, and IGF2BP3 expressions in LUSC patients were significantly different in the pathological N0/N1-3 stage (*p* < 0.05). Figure 8F showed that RBM15B expression in LUSC patients was different in the pathological M0/M1 stage (*p* < 0.05). Figure 8G indicated that expression levels of RBM15B, ZC3H13, YTHDC1, YTHDC2, YTHDF2, VIRMA, and HNRNPA2B1 in LUSC patients were significantly different in pathological TNM stages (*p* < 0.05). Figure 8H indicated that HNRNPC expression in LUSC patients was significantly different in the pathological T1/T2/T3/T4 stage (*p* < 0.01). Figure 9A showed that the expression of WTAP, RBM15, YTHDF2, RBM15B, YTHDF1, HNRNPC, IGF2BP1, VIRMA, HNRNPA2B1, and IGF2BP3 in LUAD patients was significantly positively correlated with the LUAD stem cell score (based on RNA expression data) (RNAss) (*p* < 0.05), while the expression of YTHDC2 and FTO in LUAD patients was significantly negatively correlated with the LUAD stem cell score (RNAss) (*p* < 0.05). The expression of METTL3, WTAP, RBM15, YTHDF1, HNRNPC, IGF2BP1, VIRMA, HNRNPA2B1, and IGF2BP3 had a positive relationship with the LUAD stem cell score (DNA ss) (*p* < 0.05), while the expression of METTL14 and YTHDC2 in LUAD patients had a negative relationship with the LUAD stem cell score (based on DNA methylation data) (DNA ss) (*p* < 0.05). The expression of WTAP, YTHDC2, and FTO in LUAD patients correlated positively with the LUAD microenvironment estimate score (*p* < 0.05), while the expression of METTL3, RBM15, RBM15B, YTHDC1, YTHDF1, YTHDF2, HNRNPC, ALKBH5, IGF2BP1, VIRMA, and HNRNPA2B1 in LUAD patients had a negative relationship with the LUAD microenvironment estimate score (*p* < 0.05). Figure 9B showed that the expression levels of METTL3, RBM15, YTHGC1, YTHDF1, HNRNPC, ALKBH5, IGF2BP1, HNRNPA2B1, and IGF2BP3 in LUSC patients were significantly positively associated with LUSC stem cell scores (based on RNA expression data) (RNAss) (*p* < 0.05). The expression of FTO of LUSC patients had a significant negative correlation with LUSC stem cell scores (based on RNA methylation data) (RNAss) (*p* < 0.05). The expression levels of METTL3, RBM15B, YTHDC1, YTHDC2, YTHDF2, HNRNPC, ALKBH5, IGF2BP1, HNRNPA2B1, and IGF2BP3 in LUSC patients were significantly positively associated with LUSC stem cell scores (based on DNA methylation data) (DNAss) (*p* < 0.05). The expression level of FTO in LUSC patients was negatively correlated with LUSC stem cell scores (based on DNA methylation data) (DNAss) (*p* < 0.05). The expression levels of METTL3, RBM15B, YTHDC1, YTHDF1, HNRNPC, ALKBH5, IGF2BP1, HNRNPA2B1, and IGF2BP3 in LUSC patients were significantly negatively correlated with the LUSC stromal score, immune score, and estimate score (*p* < 0.05). The expression level of RBM15 in LUSC patients had a significantly negative correlation with LUSC stromal score and estimate score (*p* < 0.05). The expression of WTAP in LUSC patients had a positive relationship with the LUSC immune score and estimate score (*p* < 0.05). The expression level of ZC3H13 had a significantly positive relation with the LUSC stromal score and estimate score (*p* < 0.05). The expression of YTHDC2 had a significantly positive relation with the LUSC immune score and estimate score (*p* < 0.05). The expression of FTO in LUSC patients was positively correlated with the LUSC stromal score, immune score, and estimate score (*p* < 0.05). The pictorial representation of m6A regulation combined with our findings is shown in Figure 10.

## Discussion

Recently, some studies demonstrated that m6A regulators are tightly associated with prognostic related to tumor types and the expression alterations of RNA methylation regulators across multiple cancer types (Liu et al., 2018; Liu et al., 2018; He et al., 2019; Li et al., 2019). m6A affects cancer progression by regulating target genes (Liu et al., 2018; Li et al., 2019). METTL3 directly promotes translation of certain mRNAs by recruiting eIF3 in human cancer cells (Lin et al., 2016). In LUAD, the higher expression of METTL3 promotes oncogene BRD4 translation by constructing a mRNA loop with EIF3(1). In acute myeloid leukemia (AML), METTL3 promotes oncogenes SP1 translation by decreasing ribosome stalling (Barbieri et al., 2017). In NSCLC, miR-33a reduced METTL3 expression and attenuated the expression levels of DNMT3A, EGFR, TAZ, and inhibited the proliferation of NSCLC cells (Du et al., 2017). Our study first identified the differential expression, Cox and immune subtype, and tumor microenvironment of m6A regulators among 33 cancer types and NSCLC, which provided a novel prospective for exploring the pivotal role of m6A regulators in pan-cancer and NSCLC.

The m6A modification is still the most significant and common modifications of human RNA molecules. It was not until around 2012 that further research on the biological functions of m6A RNA modification attracted enough attention. At that time, significant progress was made in the transcriptome profiling analysis of m6A RNA modification through high-throughput sequencing and antibody-based immunoprecipitation (Deng et al., 2018). The m6A RNA methylation plays a significant role in lung cancer (Lin et al., 2016). A study demonstrated that the m6A demethylase FTO facilitates the proliferation of NSCLC cells through upregulating the expression of USP7 (Li et al., 2019). M6A methyltransferase METTL3 activates the splicing of precursor miR-143-3p to promote lung cancer brain metastasis through VASH1 regulation (Wang et al., 2019). MiR-600 inhibited the progression of lung cancer through reducing METTL3 expression (Wei et al., 2019). ALKBH5 affected the cell growth and invasion ability of LUAD cells under intermittent hypoxia by reducing m6A modification on FOXM1 mRNA and by facilitating FOXM1 protein expression (Chao et al., 2020).

In our study, we found that the m6A methyltransferases (“writers”) ZC3H13, METTL14, VIRMA, and the m6A demethylases (“readers”) IGF2BP3 were significantly associated with overall survival of KIRC patients (*p* < 0.001). A study has demonstrated that the mRNA expression of METTL14 acts as a disease biomarker in KIRC, which has a positive relation with the overall survival of KIRC patients and negative relation with the stages of KIRC patients (Wang et al., 2019). In our study, HNRNPC was significantly correlated with LUAD patients’ overall survival (*p* = 0.001). HNRNPC served as a potential prognosis biomarker for predicting the LUAD patients’ overall survival (Zhu et al., 2020; Zhuang et al., 2020). Our study found that YTHDF2 was significantly related to LGG patients’ overall survival (*p* < 0.001). A recent study has demonstrated that YTHDF2 acts as a potential biomarker that correlated with the LGG-infiltrating immune cells (Lin et al., 2020).

In our study, we found that the m6A methyltransferases (“writers”) METTL3, ZC3H13, METTL14, VIRMA, WTAP, RBM15, RBM15B, and the m6A demethylases (“erasers”) ALKBH5, FTO, and the m6A-binding proteins YTHDC1, HNRNPC, YTHDC2, HNRNPA2B1, YTHDF1, IGF2BP3, YTHDF2, and IGF2BP1 were differentially expressed in immune subtypes. The m6A regulators were associated with the tumor microenvironment in multiple cancer types (Zhang et al., 2016; Cui et al., 2017; Bai et al., 2019; Li et al., 2019). Our study first analyzed the correlation relationship between m6A regulators expression with the tumor stem cell score across 33 cancer types. According to our results, we identified that higher the expression of m6A-related regulators, the content of immune and stromal cells was lower, and the content of tumor cells was high. Our results showed that the expression of METTL14, YTHDC1, ZC3H13, YTHDC2, and FTO in LUAD was negatively associated with the LUAD stem cell score (based on RNA expression). The higher the METTL14, YTHDC1, ZC3H13, FTO, and YTHDC2 expression level, the fewer the characteristics of tumor LUAD stem cells and higher the tumor LUAD differentiation, while the expression of METTL3, WTAP, RBM15, RBM15B, YTHDF1, YTHDF2, HNRNPC, IGF2BP1, VIRMA, HNRNPA2B1, and IGF2BP3 was positively associated with the LUAD stem cell score (based on RNA expression). The higher the expression level of METTL3, WTAP, RBM15, RBM15B, YTHDF1, YTHDF2, HNRNPC, IGF2BP1, VIRMA, HNRNPA2B1, and IGF2BP3, the more features of tumor LUAD stem cells and the lower the tumor LUAD differentiation. The expression of YTHDF1, HNRNPC, IGF2BP1, VIRMA, and HNRNPA2B1 was significantly positively correlated with the ovarian serous cystadenocarcinoma (OV) tumor stem cell score (based on DNA methylation) (DNAss). The higher expression of YTHDF1, HNRNPC, IGF2BP1, VIRMA, and HNRNPA2B1, the more characteristics of tumor OV stem cells and the lower the tumor OV differentiation. Recently, a study has demonstrated that the m6A demethylase ALKBH5 served as a negative prognostic biomarker for GBM patients and maintained the tumorigenicity of glioblastoma stem cell–like cells by activating FOXM1 expression and cell growth (Zhang et al., 2017).

Recently, a study has indicated that the evaluation of m6A methylation modification patterns within individual tumors predict stages of TME stromal activity, tumor inflammation, subtypes, genetic variation, and the survival of gastric cancer patients (Zhang et al., 2020). Higher expression of HNRNPC was significantly associated with poorer prognosis in LUAD patients (Figures 1O, 4B), which demonstrated that HNRNPC acts as oncogene that promotes the tumorigenesis of LUAD. Higher expression of IGF2BP1 was significantly associated with poorer survival of UCEC patients (Figures 1M, 4D), which indicated that IGF2BP1 plays a pivotal role in the tumorigenesis of UCEC. Lower expression of METTL14 was significantly associated with poorer prognosis in KIRC patients (Figures 1E, 4I), which showed that METTL14 acts as a tumor suppressor gene in the tumorigenesis of KIRC.

In our study, we first identified the correlation relationship between m6A regulators and anticancer drug sensitivity of CellMiner, and most m6A methylation regulators had a significantly positive association with the drug, for instance, ZC3H13 was significantly positively correlated with the drug sensitivity of selumetinib. The higher the expression of YTHDC2 and YTHDF1, the more sensitive the mediation of nelarabine, while the YTHDF2 expression was significantly negatively related to the drug sensitivity of dasatinib. The CellMiner database is a Web site of genomic and pharmacologic tools to identify drug patterns and transcript in the NCI-60 cell line (Reinhold et al., 2012). The CellMiner database that includes rapid access to and comparison of gene expression levels of 360 microRNAs, 22,379 genes, and 20,503 compounds incorporating 102 Food and Drug Administration (FDA)-approved drugs (Shankavaram et al., 2009). Our study first analyzed the relationship between m6A regulators with the drug sensitivity of anticancer drugs, which identified a novel insight for exploring tumor therapy treatment and avoiding the resistance of tumor. Selumetinib has shown promising results as a single agent or associated with conventional chemotherapy and other targeted treatments both preclinically and clinically in multiple cancers, incorporating non-small cell lung cancer, melanoma, and pediatric low-grade glioma (Weerink et al., 2017). A phase two, sequentially enrolled, multicohort, multicenter, nonrandomized, open-label research showed that dabrafenib plus trametinib provides a novel therapy with clinically meaningful antitumor activity and a potential safety profile for previous untreated BRAFV600E-mutant NSCLC patients (Planchard et al., 2017).A study has shown that YES1 acts as a predictive biomarker promotes the proliferation and progression of lung cancer and provides support for the clinical evaluation of dasatinib treatment (Garmendia et al., 2019).

In conclusion, our study identified that m6A regulators play a significant role in the immune microenvironment of 33 TCGA cancer types; meanwhile, it provided a vital insight for revealing the association between m6A regulators and anticancer drug sensitivity, which sheds a novel light on exploring mechanistic and therapeutic targets in the immune microenvironment of 33 cancer types and NSCLC.

## Data Availability

The datasets presented in this study can be found in online repositories. The names of the repository/repositories and accession number(s) can be found in the article/Supplementary Material.

## Glossary

ACC: adrenocortical carcinoma
BLCA: bladder urothelial carcinoma
BRCA: breast invasive carcinoma
CESC: cervical squamous cell carcinoma and endocervical adenocarcinoma;
CHOL: cholangiocarcinoma
COAD: colon adenocarcinoma
DLBC: lymphoid neoplasm diffuse large B-cell lymphoma
DNAss: DNA stemness score
ESCA: esophageal carcinoma
EGFR: epidermal growth factor receptor
FDA: food and drug administration
GBM: glioblastoma multiforme
HNSC: head and neck squamous cell carcinoma
KICH: kidney chromophobe
KIRC: kidney renal clear cell carcinoma
KIRP: kidney renal papillary cell carcinoma
LAML: acute myeloid leukemia
LGG: brain lower grade glioma
LUSC: lung squamous cell carcinoma
LIHC: liver hepatocellular carcinoma
LUAD: lung adenocarcinoma
MESO: mesothelioma
NSCLC: non-small cell lung cancer
OV: ovarian serous cystadenocarcinoma
PRAD: prostate adenocarcinoma
PAAD: pancreatic adenocarcinoma
PCPG: pheochromocytoma and paraganglioma
READ: rectum adenocarcinoma
RNAss: RNA stemness score
STAD: stomach adenocarcinoma
SARC: sarcoma
SKCM: skin utaneous melanoma
TGCT: testicular germ cell tumors
THCA: thyroid carcinoma
THYM: thymoma
UCEC: uterine corpus endometrial carcinoma
UVM: uveal melanoma
UCS: uterine carcinosarcoma
